# Ring-augmented Metabolic Bariatric Surgery Procedures GRADE-based International Federation for the Surgery of Obesity and Metabolic Disorders (IFSO) Position Statement

**DOI:** 10.1007/s11695-026-08556-x

**Published:** 2026-03-02

**Authors:** Kayleigh Ann Martina van Dam, Maurizio De Luca, Matteo Monami, Evert-Jan Gijsbert Boerma, Giovanni Merola, Antonio Vitiello, Ricardo Cohen, Ashraf Haddad, Khaled Gawdat, Jodok Fink, Hayssam Fawal, Amanda Belluzzi, Jan Willem Greve

**Affiliations:** 1 Department of surgery, Zuyderland Medisch Centrum, Heerlen, Netherlands; 2 Department of surgery, NUTRIM, institute for Nutrition and Translational Research in Metabolism, Maastricht University, Maastricht, Netherlands; 3 Department of Emergency, General, Bariatric and Metabolic Surgery, Ospedale Santa Maria della Misericordia di Rovigo, Rovigo, Italy; 4 Diabetology Unit, Azienda Ospedaliero-Universitaria Careggi, Florence, Italy; 5 San Giovanni di Dio Hospital, Naples, Italy; 6 Department of Advanced Biomedical Sciences, University of Naples Federico II, Naples, Italy; 7 The center for Obesity and Diabetes, Hospital Alemão Oswaldo Cruz, São Paulo, Brazil; 8 Gastrointestinal Metabolic and Bariatric Surgery Center (GBMC), Jordan Hospital, Amman, Jordan; 9 Kafrelsheikh University, Kafr ash Shaykh, Egypt; 10 Centre for Surgery, Department of General and Visceral Surgery, Centre for Obesity and Metabolic Surgery, University of Freiburg, Freiburg, Germany; 11 Makassed General Hospital, Beirut, Lebanon; 12 Department of surgery, NUTRIM, institute for Nutrition and Translational Research in Metabolism, Maastricht University, Maastricht, Netherlands

**Keywords:** ring augmentation, Metabolic Bariatric Surgery, IFSO position statement, PRISMA, Systematic Review, meta-analysis, GRADE methodology

## Abstract

In this position statement, the International Federation for the Surgery of Obesity and Metabolic Disorders (IFSO) highlights the evaluation of ring-augmented procedures in Metabolic Bariatric Surgery (MBS). A GRADE-based systematic review and meta-analysis were conducted to assess their effectiveness and safety. The analysis included 28 studies with 6939 patients. The findings demonstrated that ring augmentation in bariatric procedures may provide superior or comparable weight loss outcomes compared to standard procedures, although results were heterogenous and evidence quality varied. Ring-related complications and removal rates were generally low. While further high-quality, long-term studies are needed, current evidence cautiously supports selective use of ring augmentation in both primary and revisional MBS, particularly for Roux-en-Y Gastric Bypass (raRYGB).

## Introduction / Background

Metabolic Bariatric Surgery (MBS) remains the most effective long-term treatment in patients with obesity. Literature has consistently demonstrated MBS to be effective in achieving substantial weight loss and in improving obesity-related medical problems, such as type 2 diabetes mellitus and hypertension [1–4]. According to the 8th IFSO Global Registry Report 449,815 primary bariatric procedures were performed worldwide in 2023, highlighting the international scale and importance of surgical interventions in the treatment of obesity [5]. Among these primary procedures, the Sleeve Gastrectomy (SG), Roux-en-Y Gastric Bypass (RYGB), and One Anastomosis Gastric Bypass (OAGB) have become the most commonly performed procedures [2, 3, 6].

Despite the overall of success of MBS, recurrent weight gain and long-term complications remain a concern for patients [7, 8]. Recurrent weight gain is reported in 3.9% to 49% of patients within the first five years after surgery, depending on procedure type and definition applied [8–10]. To address the recurrent weight gain challenge, ring augmentation has been proposed as a surgical strategy to enhance long-term weight maintenance [11, 12]. Ring augmentation is the placement of a silicone ring around the gastric pouch or sleeve to enhance satiety during meals without causing constant mechanical restriction. For the ring augmented RYGB (raRYGB) it is for example hypothesized that when the pouch expands against the ring the mechanical interaction triggers neural signals that promote a feeling of satiety [13]. To avoid confusion with adjustable gastric banding, the term ring augmentation is used instead of the commonly used banded, as it more accurately describes the placement of a non-adjustable silicone ring [14].

Ring augmentation is not an entirely new concept as various devices have been used in attempts to enhance weight loss durability. Early and mid-term studies suggest that ring augmented procedures, such as raRYGB, may improve weight loss and prevent long-term recurrent weight gain compared to standard procedures [9, 11, 12]. Ring augmentation has also been introduced in revisional and conversional MBS, for instance following SG [15, 16]. However, the existing evidence on ring augmented procedures remains heterogenous with variations in ring type, surgical techniques and reported outcomes.

With the growing use of ring augmentation in bariatric surgery, there is a need for a clear, evidence-based framework to guide clinical practice. Therefore, this position statement aims to categorize ring augmented MBS and to review current evidence on outcomes such as weight loss, complication rate and ring removal rate.

## Methods

### Position Statement Methods

This ring-augmented Metabolic Bariatric Surgery procedures position statement of the International Federation for the Surgery of Obesity and Metabolic Disorders (IFSO) is aimed at assessing ring augmented MBS. To develop the present PS, IFSO nominated experts (all co-authors of the present paper) in the field of obesity management. All members reported a declaration of potential conflicts of interest (COI), which were collectively discussed to determine their relevance. For all the involved members, COI were considered trivial or irrelevant. The panel of experts decided to adopt the Grading of Recommendations, Assessment, Development, and Evaluation (GRADE) as described below and to formulate any recommendation exclusively on the results of meta-analyses of Randomized Clinical Trials (RCT).

## PICO Framework

This review was structured using the PICO (Population, Intervention, Comparison, Outcome) framework. The population included adults (*≥* 18 years) with obesity undergoing MBS. The intervention consisted of ring-augmented procedures, including RYGB, SG and OAGB. The comparison group underwent standard versions of these procedures. The outcomes of interest were postoperative weight loss, measured as either %TWL of %EWL, and complication rates including ring removal. A secondary PICO was applied to identify and analyze studies reporting outcomes of conversional bariatric procedures involving ring augmentation. This methodological approach enhanced the transparency, reproducibility, and global applicability of the PICOs of the Position Statement [17].

### PICO Definitions

- PICO 1 – In adults with obesity undergoing primary MBS, do ring-augmented procedures result in greater long-term weight loss and acceptable complication rates compared to standard procedures?
- PICO 2 – In adults undergoing conversional/revisional MBS, does the addition of ring augmentation improve weight loss outcomes without affecting the overall safety compared to standard procedures?

### Literature Search and SystematicReview

A comprehensive literature search was conducted to identify studies reporting outcomes of ring augmented MBS procedures. The search was done in accordance with the Preferred Reporting Items for Systematic Reviews and Meta-Analyses (PRISMA) guidelines [18]. Electronic databases including PubMed and the Cochrane Library were searched up to May 2025. Search terms encompassed standard bariatric procedures (e.g. ‘’*RYGB’’*,* ‘’SG’’*,* ‘’OAGB”)* and ring augmentation terminology (e.g. *‘’ring-augmented’’*,* ‘’banded’’*,* ‘’ring’’*,* ‘’MiniMizer’’).* The complete search strategy is presented in appendix 1. Additionally, reference lists of relevant reviews and included studies were manually screened to identify further eligible publications.

### Inclusion and Exclusion Criteria

Studies were eligible if they reported outcomes of bariatric procedures involving the placement of a non-adjustable ring around the gastric pouch or sleeve. Only full-text articles published in peer-reviewed journals were considered. To ensure comparability, only studies that included a control group undergoing standard procedure were eligible for the primary procedures. Descriptive studies reporting outcomes exclusively for ring-augmented procedures without a comparator group were excluded. For revisional procedures the studies without a comparator group were included. Additionally, studies were excluded if they focused on banded procedures such as adjustable gastric banding or if they did not explore or fully reported data concerning at least one of the considered outcomes (i.e. weight loss, complication rate and ring removal rate).

### Data Extraction

Duplicates were identified and removed using RefWorks (Clarivate, United States). Data were extracted and included information about the study design and methodology, participant demographics, details about ring-augmented and standard procedures, weight loss outcomes and complication outcomes. Especially information regarding surgical technique, follow-up duration, total weight loss (%TWL) and excess weight loss (%EWL), and complications were extracted. Data extraction was performed independently by two reviewers, with discrepancies resolved by consensus.

### Quality Assessment of Included Studies

The risk of bias was assessed using the Cochrane recommended tool to determine risk of bias in RCTs [19]. The risk of bias was described and evaluated in seven specific domains: random sequence generation, allocation concealment, blinding of participants and personnel, blinding of outcome assessment, incomplete outcome data, selective reporting, and other biases. The results of these domains were graded as ‘’low’’ risk of bias, ‘’high’’ risk of bias, or ‘’uncertain’’ risk of bias. Observational studies (retrospective and prospective cohort studies) were evaluated using the Newcastle-Ottawa Scale (NOS). Two researchers (KvD and JWG) independently assessed the risk of bias in individual studies, with discrepancies resolved through discussion and if necessary, by a third researcher.

### GRADE Assessment of the Quality of Retrieved Evidence

The overall quality of retrieved studies has been evaluated and rated (“very low, low, moderate, or high) using GRADEpro software (https://gdt.gradepro.org/) [20].

### Statistical Analysis

All the analyses were separately performed based on the design of the included studies (i.e. randomize and non-randomized studies). Meta-analyses were performed using R (version 4.5.0) with the meta package. Continuous variables were reported as means with standard deviations (SD) or as medians with interquartile ranges (IQR). Categorical outcomes were expressed as proportions. Due to methodological differences %TWL and %EWL were stratified and analyzed separately. Likewise, due to differences in surgical technique outcomes were also stratified by primary procedure type (RYGB, SG, and OAGB). Revisional procedures were evaluated separately for weight loss outcomes.

Mean difference (MD) was used as the measure of effect for all continuous outcomes. A random-effects model was used. Fixed-effect models were computed for comparison but not reported when heterogeneity was high. This heterogeneity among studies was assessed using the Higgings I^2^ statistic with I^2^ values > 50% indicated substantial heterogeneity. In such cases, random-effects models were prioritized. Studies that reported mean values without accompanying SD or confidence intervals were excluded from meta-analysis as the necessary variance estimates could not be derived. In addition, leave-one-out sensitivity analyses were conducted to assess the impact of individual studies on pooled outcomes and heterogeneity. Forest plots were developed to visualize the analyses. Publication bias was assessed using Egger’s test. A p-value of < 0.05 was considered statistically significant.

## Results

### Literature Search

**Fig. 1:**
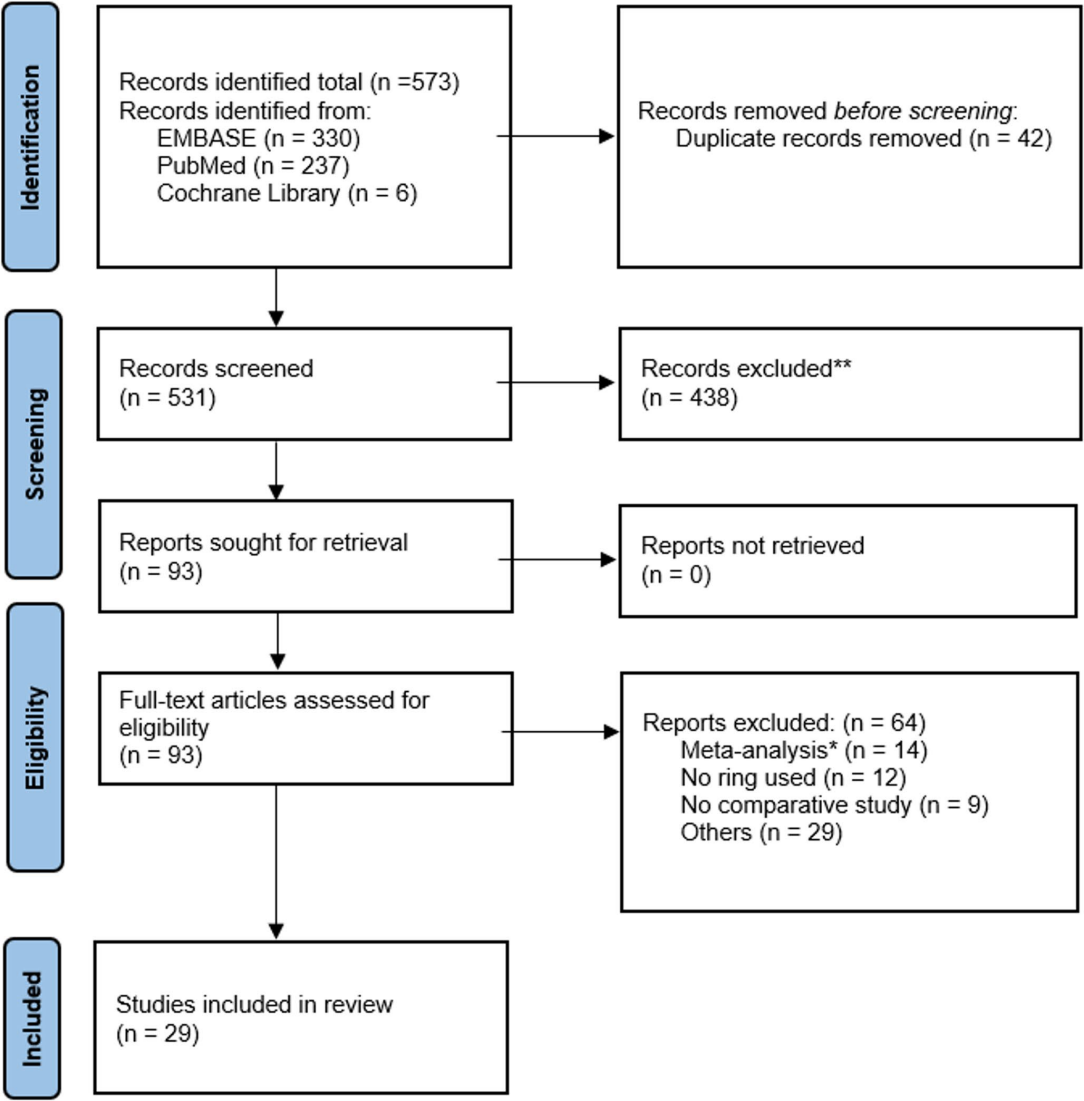
PRISMA flowchart for literature screening and inclusion. * in case of meta-analysis, the original studies were screened for eligibility

Using the described search strategy, initially 573 studies were identified. After removing 42 duplicates the titles and abstract were screened for 531 records. Full text articles for 93 eligible studies were screened. A total of 64 studies were excluded due to lack of ring augmentation data, non-comparative design or insufficient outcome reporting (Fig. 1). In total 29 articles involving 6999 patients were included. Key study characteristics of the studies are summarized in Tables 1 and 2.

**Table 1:** Outcomes of observational studies included regarding primary Metabolic Bariatric Surgery

Author	Study design	Procedures (*n*)	Ring type	Follow-up (years)	Weight loss outcome (%TWL/%EWL)	(Major) complications	Ring-related complications
Skidmore et al., 2022 [22]	Prospective case control	raRYGB (116) vs. RYGB (368)	MiniMizer	1.5	65.7% *±* 37% EWL 62.2% *±* 30% EWL	NA	21.7%
Tsai et al., 2022 [23]	Retrospective cohort study	raRYGB (55) vs. RYGB (55)	Silastic Fobi ring	5	28.3% *±* 10% TWL 30 *±* 9.7% TWL	NA	16.3%
Ferreira et al., 2024 [24]	Retrospective cohort study	raRYGB (409) vs. RYGB (449)	Silicone ring	10	Na	13.36% vs. 8.68%	9.53%
Jense et al., 2022 [13]	Retrospective cohort study	raRYGB (191) vs. RYGB (184)	Ventriculoperitoneal drain	5	32.6% *±* 9.1% TWL 27.6% *±* 9.02% TWL	7.9% vs. 4.3%	4.3%
Lemmens, 2017 [25]	Prospective cohort study	raRYGB (178) vs. RYGB (254)	GaBP ring	5	74% *±* 15.1% EWL 65.2% *±* 20% EWL	9% vs. 3.1%	2.8%
Bhandari et al., 2016 [26]	Retrospective cohort study	raRYGB (64) vs. RYGB (101)	GaBP ring	2	71.46% *±* 7.78% EWL 60.76% *±* 8% EWL	NA	NA
Awad et al., 2012 [27]	Retrospective cohort study	raRYGB (260) vs. RYGB (218)	PTFE and Goretex	10	82% EWL vs. 63% EWL	NA	NA
Heneghan et al., 2014 [28]	Matched cohort analysis	raRYGB (134) vs. RYGB (134)	Silicone ring	2	58.6% EWL vs. 51.4% EWL	Early: 19.4% vs. 19.4% Late 10.4% vs. 13.4%	1.5%
Jense et al., 2025 [29]	Prospensity score matched analysis	raRYGB (296) vs. RYGB (296)	MiniMizer	5	31.5% *±* 8.8% TWL 28% *±* 9.7% TWL	3.4% vs. 2%	2.4%
Bhandari et al., 2019 [34]	Retrospective cohort study	raRYGB (82) vs. OAGB (90)	GaBP ring	5	30.5% *±* 8.2% TWL 34.7% *±* 7% TWL	NA	NA
Fink et al., 2024 [35]	Observational follow-up after RCT	raSG (42) vs. SG (40)	MiniMizer	5	31.6% *±* 13.6% TWL 27.4% *±* 12.6% TWL	NA	2%
Hany et al., 2022 [36]	Retrospective cohort study	raSG (132) vs. SG (1279)	MiniMizer	4	43.45% *±* 7.68 TWL 42.56% *±* 11.65% TWL	0.8% vs. 0.46%	0%
Bhandari et al., 2019 [38]	Prospective cohort study	raSG (68) vs. SG (152)	GaBP ring	5	83% EWL vs. 53% EWL	NA	0%
Fink et al., 2019 [39]	Retrospective matched-pair analaysis	raSG (51) vs. SG (51)	MiniMizer	5	37.6% *±* 8.5% TWL 29.5% *±* 12.9% TWL	NA	11.8%
Karcz et al., 2014 [40]	Retrospective matched-pair analysis	raSG (25) vs. SG (25)	MiniMizer	1	58.36% EWL vs. 58.02% EWL	NA	8%
Albalkiny et al., 2021 [41]	Prospective cohort study	raSG (49) vs. SG (50)	PTFE ring	3	70.5% *±* 5% EWL 64% *±* 16% EWL	early: 18.4% vs. 16% Late: 32.5% vs. 24%	NA
Salvi et al., 2020 [42]	Retrospective cohort study	raSG (68) vs. OAGB/MGB (55)	GaBP ring	6	84% EWL vs. 70% EWL	NA	NA
Quint et al., 2024 [44]	Retrospective cohort study	raOAGB (24) vs. OAGB (43) vs. SG (44)	Unclear	1	65.3% *±* 18.4% EWL 59.4% *±* 25.1% EWL 63.5% *±* 20% EWL	4.1% vs. 0% vs. 4.5%	NA
Elhoofy et al., 2024 [45]	Prospective cohort study	raOAGB (43) vs. OAGB (44)	MiniMizer	3	NA	Early: 16.3% vs. 13.% Late: 25.6% vs. 22.7%	4.7%

raSG = ring augmented sleeve gastrectomy, SG = sleeve gastrectomy, raRYGB = ring augmented roux-en-y gastric bypass, RYGB = roux-en-y gastric bypass, raOAGB = ring augmented one-anastomosis gastric bypass, OAGB = one-anastomosis gastric bypass,

**Table 2:** Outcomes of RCTs included regarding primary Metabolic Bariatric Surgery

Author, year [ref]	Study design	Number of patients	Ring type	Follow-up (years)	Weight loss outcomes	(Major) complications	Ring removal
Pullman et al., 2023 [31]	RCT	raRYGB (56) vs. SG (58)	Silastic ring	7	26.2% TWL vs. 13.4% TWL	Early: 3.6% vs. 8.6% Late: 16.1% vs. 10.3%	5.4%
Murphy et al., 2022 [32]	RCT	raRYGB (53) vs. SG (55)	Silastic ring	5	69.7% *±* 24.9% EWL 40.3% *±* 20.3% EWL	Early: 3.6% vs. 8.6% Late: 19.6% vs. 12.1%	0%
Zarate et al., 2013 [30]	RCT	raRYGB (30) vs. RYGB (30)	Polypropylene mesh	5	61.6% *±* 19.6% EWL 59.8% *±* 15.9% EWL	NA	3.33%
Murphy et al., 2018 [33]	RCT	raRYGB (56) vs. SG (58)	Silastic ring	1	84.2% *±* 26.2% EWL 70.2% *±* 19.4% EWL	16.3% vs. 12.1%	1.8%
Fink et al., 2020 [37]	RCT	raSG (47) vs. SG (47)	MiniMizer	3	73.9% *±* 21.3% EWL 62.3% *±* 21.5% EWL	6.6% vs. 4.3%	2.2%
Cazzo et al., 2020 [43]	RCT	raOAGB (16) vs. OAGB (17)	Silastic ring	1	27.5% *±* 6.6% TWL 30.5% *±* 7.1% TWL	NA	NA

Out of these 29 retrieved studies, 7 were RCT and 22 were observational studies. Among the RCTs the reported mean age for patients undergoing ring augmented procedures ranged from 35.9 to 48.4 years, while for standard MBS it ranged from 33.8 to 46.7 years. The proportion of female patients was similar, ranging between 41% and 90%. BMI at baseline for ring augmented procedures ranged from 42.2 to 51 kg/m^2^ and from 41.7 to 50.7 kg/m^2^ for standard MBS.

In the observational studies the mean age of patients undergoing ring-augmented procedures ranged from 34.4 to 47.6 years, while for standard MBS it was between 34.3 and 49.2 years. The percentage of female patients varied between 33.3 and 85.1% and this range distribution was comparable between ring augmented and standard procedures. Regarding baseline BMI patients with ring augmented procedures had a BMI ranging between 41 and 56.1 kg/m^2^, while patients with standard procedures had a BMI between 39.8 and 57 kg/m^2^.

## Studies on Primary Surgery with Ring Augmentation

### Ring-augmented Roux-en-Y Gastric Bypass (raRYGB)

A total of fourteen studies reported outcomes of raRYGB including a total of 4330 patients. Of these, ten studies directly compared raRYGB to standard RYGB and were included in the analysis [12, 21–29]. The remaining four studies compared raRYGB to other procedures such as SG or OAGB [30–33]. Due to the inability to draw conclusions about the relative effectiveness of raRYGB versus its standard counterpart these studies were excluded. For the included studies, the ring types varied and included MiniMizer, Silastic rings and GaBP ring. Follow-up durations ranged from 1.5 to 10 years, and sample sizes ranged from 60 to 592 patients. Across the studies, weight loss outcomes were assessed using both %EWL and %TWL. Six studies reported %EWL and three reported %TWL outcomes while the study of Ferreira et al. did not have weight loss related outcomes [23]. Across the studies, the raRYGB had more %EWL or %TWL than the standard procedure.

#### Weight loss (%EWL)

**Fig. 2:**
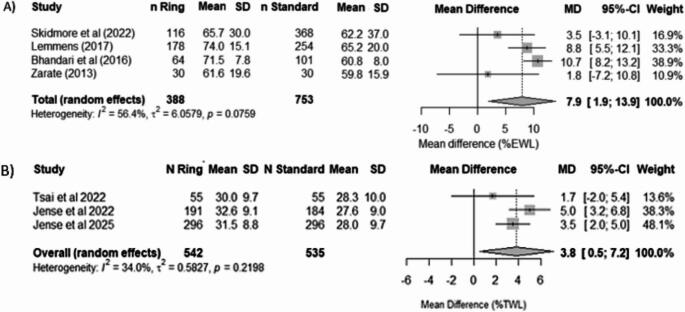
Forest plot for %EWL and %TWL comparing raRYGB versus standard procedures. **A**) %EWL with observational studies, **B**) %TWL with observational studies

Out of five studies initially considered, two were excluded due to insufficient statistical data [31, 32]. For the meta-analysis three of the remaining studies were observational studies and one was a RCT [21, 24, 25, 29]. These four studies reported %EWL outcomes with a sample-size-weighted mean follow-up duration of 3.1 years, with individual follow-up durations varying between 1.5 and 5 years. The pooled analysis showed a benefit of ring augmentation with a mean difference (MD) of 7.9% (95% CI 1.9–13.9) as shown in Fig. 2. Heterogeneity was high (I^2^ = 56.4%). Sensitivity analysis through leave-one-out showed that the pooled effect remained stable as the inclusion of individual studies resulted in pooled mean %EWL between 67 and 71.3% (appendix 2). In addition, heterogeneity remained substantial with I^2^ values of at least 80%. All of this indicates that no single study disproportionately influenced the overall result. Regarding publication bias, Egger’s test showed an intercept of -2.35 (*p* = 0.14), suggesting no strong evidence of small-study effects.

#### Weight Loss (%TWL)

No RCTs reported information on %TWL. This outcome was reported in three observational studies, all with a mean follow-up of five years [12, 22, 28]. The pooled mean difference showed a significant between-group difference (8.84%, 95% CI 5.82–11.87) (Fig. 2). Heterogeneity was low with a I^2^ value of 34%. For the sensitivity analysis, exclusion of the study by Jense et al. led to a major reduction in heterogeneity (I^2^ = 0%) [12]. However, the narrow range of the pooled mean across all analyses (29.5% − 30.1%) supports the stability of the overall effect and confirms that no singly study, including Jense et al., disproportionately influenced the result (appendix 2). Egger’s test showed no evidence of small-study effects (t = -0.55, *p* = 0.68).

### Ring-augmented Sleeve Gastrectomy (raSG)

Eight studies, 1 RCT and 7 observational, reported outcomes of raSG with variations in study design, ring type and follow—up duration which ranged from 1 to 5 years [34–41]. The sample sizes ranged from 50 to 1411 patients. Seven studies directly compared raSG to standard SG [34–40]. Only one study compared raSG to another procedure, namely to OAGB which was then excluded [41]. The majority reported weight loss outcomes regarding %EWL [36, 37, 39, 40]. Only three studies used %TWL to assess weight loss outcomes [34, 35, 39]. All studies showed higher weight loss percentages for the raSG group as shown in Table 1. However, the study of Karcz et al. had a minimal superior result with only a 0.34% difference in EWL [39].

#### Weight Loss (%EWL)

For %EWL, a meta-analysis was not performed due to the limited number of studies with different designs. The observational study of Albalkiny et al. with three year follow-up reported 70.5% EWL in the raSG group versus 64% in the standard SG group [40]. The study of Fink et al. was a RCT with three year follow-up with 73.9% EWL in the raSG group and 62.3% in the standard SG group [36].

#### Weight loss (%TWL)

**Fig. 3:**
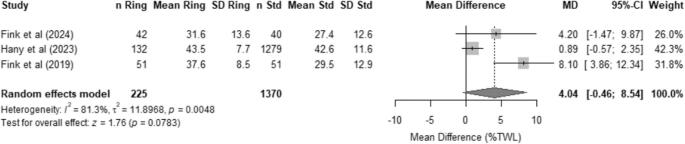
Forest plot for %TWL comparing raSG versus standard procedures

Three observational studies reported information on this outcome with a sample-size-weighted mean follow-up of 4 years (range 1–5 years) and were included in a formal meta-analysis [34, 35, 39]. The meta-analysis showed a not significant trend in favor of raSG with a pooled mean difference of 4.04% (95% CI -5.31–13.38, *p* = 0.2040) (Fig. 3). Heterogeneity was high with a I^2^ value of 81.3%. The sensitivity analysis showed pooled mean %TWL values ranging from 34.9% to 40.6% (appendix 2). The pooled mean remained within a relatively narrow range which supports the consistency of improved weight loss with ring augmentation. Egger’s test showed an intercept of 3.03 with a non-significant with *p* = 0.356 suggesting no evidence of small-study effects or publication bias.

### Ring-augmented One-anastomosis Gastric Bypass (raOAGB)

Three studies, 1 RCT and 2 observational reported outcomes of raOAGB compared to standard OAGB [42–44]. A total of 231 patients were included with follow-up periods between 1 and 3 years. The study of Elhoofy et al. did not report weight loss outcomes and was therefore excluded from the analyses [44]. The RCT by Cazzo et al. showed a slightly lower %TWL at 1 year in the raOAGB group (27.5% vs. 30.5%). The observational study of Quint et al. reported higher %EWL at 1 year in the raOAGB group (65.3%) compared to standard OAGB (59.4%). Due to the limited number of studies with adequate data of weight loss outcomes no meta-analysis was performed for raOAGB.

## Studies on Revisional/Conversional Surgery with Ring Augmentation

Four studies, 1 RCT and 3 observational, have reported outcomes of ring augmentation in revisional MBS as shown in Table 3 [15, 45–47]. These studies varied in design and type of procedure. Two studies focused on revisional procedures, with pouch revision and resleeve, while the other two focused on conversional procedures. All of these studies used %TWL as weight loss outcome and three provided cumulative %TWL, reflecting total weight loss from the initial procedure. The studies had a sample-size-weighted mean follow-up of two years (range 1–3 years). Reported cumulative %TWL values ranged from 26.9% to 32.9%, indicating a generally consistent trend across studies. However, only one study directly compared ring-augmented revisional procedure to its standard revisional counterpart [45].

**Table 3:** Outcomes of the studies included regarding revisional Metabolic Bariatric Surgery

Author	Study design	Procedures (*n*)	Ring type	Follow-up (years)	Weight loss outcome (%TWL/%EWL)	(Major) complications	Ring removal
van Dam et al., 2024 [16]	Retrospective cohort study	SG to raRYGB (50)	MiniMizer	1	17.8% TWL 32% cumulative TWL	16%	6%
Hany et al., 2023 [46]	RCT	ra-reSleeve (28) vs. resleeve (31)	MiniMizer	2	44.2% TWL 42.2% TWL	16% vs. 12%	4%
Bhandari et al., 2022 [47]	Retrospective cohort study	SG to raRYGB (62)	GaBP ring	3	14.5% TWL 26.9% cumulative TWL	NA	0%
Boerboom et al., [48]	Retrospective cohort study	RYGB to raRYGB (79)	MiniMizer	2	26% cumulative TWL	32.9%	23%

### Ring Related Complications

Ring-related complications and the removal rate were reported in most of the included studies [12, 15, 21–24, 27–31, 34–39, 44–47]. Overall, removal rates ranged from 0% to 21.7% with most studies (13/21) reporting rates of 5% and less (Tables 1 and 2). In total, 129 rings were removed among 2041 patients during follow-up. The highest removal rate was observed in a study on raRYGB of Skidmore et al. with 21.7% [21]. On the other hand, four studies reported no removals at all resulting in a removal rate of 0% [31, 35, 37, 46]. Besides ring removal, there were a few studies with reported ring-related complications [24, 26]. In addition to a 2.8% removal rate, the study of Lemmens also reported a 3.4% replacement rate [24]. One study, namely by Awad et al., demonstrated a 1.2% migration rate after using the PTFE ring, but none after using the Goretex ring [26].

**Fig. 4:**
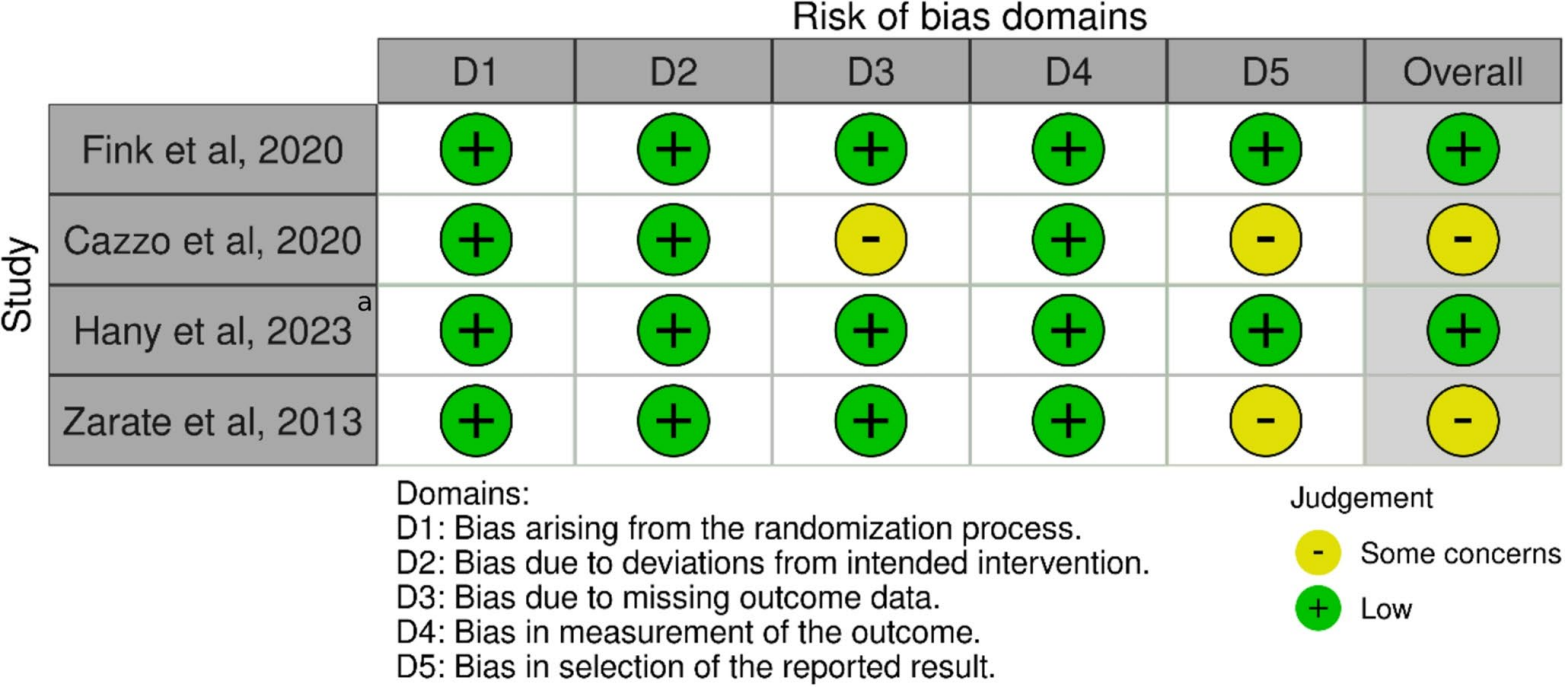
Cochrane Risk of Bias assessment of RCTs for both primary and revisional proceduresarevisional MBS

Due to lack of direct comparisons and insufficient statistical data a meta-analysis could not be performed. However, there are several observational studies with large patient cohorts and long follow-up time with comparable complication rates between raRYGB and RYGB, such as the study by Jense et al. (3.4% vs. 2%) [12, 23].

### GRADE Evaluation of Retrieved Evidence

On the basis of retrieved studies (RCTs) the overall quality of evidence for raRYGB was rated as ‘’moderate’’ and ‘’low’’ for efficacy and safety outcomes, respectively (Table 4).

**Table 4:** GRADE evaluation of retrieved evidence (RCT) for raRYGB

Certainty assessment							Summary of findings				
							Study event rates (%)			Anticipated absolute effects	
Participants (studies) follow-up	Risk of bias	Inconsistency	Indirectness	Imprecision	Publication bias	Overall certainty of evidence	With placebo	With IFSO LSI add-on	Relative effect (95% CI)	Risk with placebo	Risk difference with IFSO LSI add-on
%EWL											
222 (2 RCTs)	Serious^a^	Serious^b^	Not serious	Serious^c^	Very strong association	⨁⨁⨁◯ Moderate^a, b,c^	113	109	-	113	MD 21.69 higher (6.59 higher to 36.78 higher)
Adverse events (early and late)											
336 (3 RCTs)	Serious^a^	Not serious	Not serious	Serious^c^	None	⨁⨁◯◯ Low^a, c^	28/171 (16.4%)	35/165 (21.2%)	OR 1.38 (0.79 to 2.39)	28/171 (16.4%)	49 more per 1000 (from 30 fewer to 155 more)

*CI* confidence interval, *MD* mean difference, *OR* odds ratio

^a^ open-label study, ^b^ high heterogeneity (I^2^ >50%), ^c^ very small sample size and/or data coming from only one study

### Quality of Assessment

The risk of bias assessment for included RCTs showed that two out of four [36, 45] were rated as having low risk of bias across all domains (Fig. 4). The RCT by Cazzo et al., was rated as having ‘some concerns’ in domains related to outcome measurement and reporting [42]. The observational studies were assessed using the Newcastle-Ottawa Scale which revealed that the majority of the studies scored between 7 and 9 stars, indicating low to moderate risk of bias (Table 5) [12, 21–25, 27, 28, 34, 35, 37–41, 43, 44]. Only one study achieved the highest score of 9 stars [34]. Only the study by Awad et al. received a score of 4 stars [26].

**Table 5:** Newcastle – Ottawa Quality Assessment Scale for observational studies

Study	Selection	Comparability	Outcome	Total stars
Skidmore et al., 2022	★★★★	★	★★	7
Tsai et al., 2022	★★★★	★	★★★	8
Ferreira et al., 2024	★★★	★	★★	6
Jense et al., 2022	★★★★	★	★★★	8
Lemmens et al., 2017	★★★★	★	★★★	8
Bhandari et al., 2016	★★★★	★	★★	7
Awad et al., 2012	★★		★★	4
Heneghan et al., 2014	★★★★	★	★★	7
Jense et al., 2025	★★★★	★★	★★	8
Bhandari et al., 2019	★★★★	★	★★★	8
Fink et al., 2024	★★★★	★★	★★★	9
Hany et al., 2023	★★★	★★	★★★	8
Fink et al., 2019	★★★★	★	★★	7
Karcz et al., 2014	★★★★	★	★★	7
Albalkiny et al., 2021	★★★★	★	★★	7
Quint et al., 2024	★★★★		★★	6
Elhoofy et al., 2024	★★★★	★	★★	7

## Discussion

This position statement reviewed the current evidence on ring augmented MBS procedures, focusing on efficacy, safety and applicability across procedures. The available data, particularly for raRYGB, suggest a potential for greater weight loss compared to standard RYGB. However, these findings are limited by substantial heterogeneity in ring type, study design, follow-up duration, and outcome reporting, as well as by low-to-moderate evidence quality.

Most included studies directly compared raRYGB to standard RYGB. However, a subset of studies also evaluated raRYGB against other commonly performed procedures such as SG and OAGB. These were excluded from the meta-analysis due to lack of direct comparability. raRYGb being superior or inferior to SG or OAGB does not imply superiority over standard RYGB. Therefore, only direct comparisons of the same procedure were used for quantitative synthesis. Some RCTs and large observational cohorts support improved outcomes without increased complications, however the certainty of evidence remains insufficient to make strong recommendations, especially for raSG and raOAGB where data are sparse.

These findings are in accordance with several previous meta-analyses focused only on raRYGB [11, 48, 49]. The present meta-analysis performed for developing this IFSO position statement consists of the inclusion of a higher number of studies and the evaluation of procedures other than raRYGB. Unfortunately, data on raSG and raOAGB remains limited, preventing reliable recommendations. Preliminary observational data regarding efficacy and safety data seems to have a trend favoring ring augmentation. This positive trend is comparable to some meta-analysis on banded SG, which found a lower BMI and more %EWL [50–52]. Additionally, a systematic review by Gupta et al. assessed %TWL instead of %EWL and reported higher weight loss for banded SG [53]. This lack of studies limits the generalizability of findings for all ring-augmented procedures.

In conversional or revisional surgery ring augmentation the cumulative %TWL is comparable with the %TWL after a primary standard RYGB of 27.2–30.6% [54–56]. This suggests ring augmentation may be a valuable strategy for patients with suboptimal clinical outcomes after primary MBS. However, these considerations are still speculative since they are based on a small number of heterogenous studies with different follow-up durations.

### Limitations

Some limitations must be acknowledged when interpreting the findings of this position statement. One major challenge is the inconsistency in reporting weight loss outcomes across studies, with some using percentage excess weight loss (%EWL) and others percentage total weight loss (%TWL). This variation complicates direct comparisons and limits the synthesis of results in meta-analyses.

Additionally, the availability of comparative studies differs between procedures. While raRYGB has been relatively well studied, raSG and especially raOAGB lack sufficient high-quality comparative studies. This makes it difficult to draw reliable conclusions for these procedures.

Furthermore, the included studies show considerable heterogeneity in terms of design, ring types, surgical techniques, and follow-up durations, which adds complexity to the interpretation of pooled outcomes and limits the generalizability of the results.

## Conclusion

Ring augmented Metabolic Bariatric Surgery appears to be a promising but not yet fully validated strategy to enhance long-term weight loss outcomes. The most consistent evidence exists for ring augmented RYGB, which shows superior weight loss outcomes compared to standard RYGB. For ring augmented SG and OAGB, evidence is limited, heterogeneous, and requires cautious interpretation. In revisional MBS, preliminary findings are encouraging, but current evidence is insufficient for routine recommendations. Further high-quality, long-term RCTs with standardized outcome reporting are essential before broader adoption.

In revisional MBS ring augmentation shows encouraging results with cumulative %TWL comparable to those of primary procedures. Importantly, the generally low complication and removal rates support the safety of ring augmentation when applied selectively. However, to validate and strengthen these findings and enable standardized outcome reporting high quality, long-term comparative randomized controlled trials are needed.

### Recommendations of the IFSO Ring Augmented Metabolic Bariatric Surgery Procedures Task Force

1. In primary MBS, ring augmentation may be considered selectively, particularly for patients at high risk of recurrent weight gain, but only after discussion of limited long-term evidence.
2. In revisional or conversional MBS, the addition of a ring should not be routinely recommended. When considered, it should be performed only in specialized centers with structured follow-up, ideally within clinical trials or registries.
3. Ring-augmented procedures in revisional settings should be limited to prospective data collection, preferably under IRB approval.
4. Further well-designed, long-term RCTs are strongly needed to clarify the role of ring augmentation, particularly in revisional and conversional surgery, where evidence is currently sparse and of low certainty.

## Data Availability

No datasets were generated or analysed during the current study.

## Appendix 1. Literature Search

**Table 1:** List of search terms used

Population	Intervention	Comparison	Outcome
Metabolic Bariatric Surgery	Ring augmented	Standard	Total weight loss
Bariatric surgery	Ring Augmentation		Weight loss
Sleeve gastrectomy	Ring		Complications
Roux-en-y Gastric Bypass	Banded		Removal
Gastric Bypass	MiniMizer		
Gastric Sleeve	Silastic Ring		
One anastomosis gastric bypass			
Single anastomosis gastric bypass			

## Appendix 2. Leave-one-out Sensitivity Analysis

**Table 1:** Leave-one-out analysis for raRYGB regarding %TWL

Excluded Study	Pooled Mean %TWL	95% CI (Lower – Upper)	I² (%)
Tsai et al., 2022	30.1	27.8–32.4	35.4
Jense et al., 2022	29.5	26.2–32.8	0
Jense et al., 2025	29.8	25.5–34.1	59.6

**Table 2:** Leave-one-out analysis for raSG regarding %TWL

Excluded Study	Pooled Mean %TWL	95% CI (Lower – Upper)	I² (%)
Fink et al., 2024	40.6	34.9–46.3	94.6
Hany et al., 2023	34.9	29–40.7	83.8
Fink et al., 2019	37.7	26.1–49.3	96.5

## References

[1] Jakobsen GS, Småstuen MC, Sandbu R, et al. Association of bariatric surgery vs medical obesity treatment with long-term medical complications and obesity-related comorbidities. JAMA. 2018;319(3):291–301. 29340680 10.1001/jama.2017.21055 PMC5833560

[2] Perdomo CM, Frühbeck G, Frühbeck J et al. Clinical perspectives, eligibility, and success criteria for bariatric/metabolic surgery. In: Frühbeck G, editor. *Obesity and Lipotoxicity*. 2024. pp. 677–95. 10.1007/978-3-031-63657-8_23 39287869

[3] Chang SH, Stoll CR, Song J, et al. The effectiveness and risks of bariatric surgery: an updated systematic review and meta-analysis, 2003–2012. JAMA Surg. 2014;149(3):275–87. 24352617 10.1001/jamasurg.2013.3654 PMC3962512

[4] Aderinto N, Al Omran Y, Al-Mazrou AM, et al. Recent advances in bariatric surgery: a narrative review of weight loss procedures. Ann Med Surg (Lond). 2023;85(12):6091–104. 38098582 10.1097/MS9.0000000000001472 PMC10718334

[5] International Federation for the Surgery of Obesity and Metabolic Disorders (IFSO). 8th IFSO Global Registry Report 2024. Oxford: Dendrite Clinical Systems. 2024 [cited 2025 Jun 8]. Available from: https://www.ifso.com/pdf/8th-ifso-registry-report-2024-latest-new.pdf

[6] Hu Z, Sun J, Li R, et al. A comprehensive comparison of LRYGB and LSG in obese patients including the effects on QoL, comorbidities, weight loss, and complications: a systematic review and meta-analysis. Obes Surg. 2020;30(3):819–27. 31834563 10.1007/s11695-019-04306-4 PMC7347514

[7] Noria SF, Grantcharov T, Switzer NJ, et al. Weight regain after bariatric surgery: scope of the problem, causes, prevention, and treatment. Curr Diab Rep. 2023;23(3):31–42. 36752995 10.1007/s11892-023-01498-z PMC9906605

[8] El Ansari W, Elhag W, et al. Weight regain and insufficient weight loss after bariatric surgery: definitions, prevalence, mechanisms, predictors, prevention and management strategies, and knowledge gaps—a scoping review. Obes Surg. 2021;31:1755–66. 33555451 10.1007/s11695-020-05160-5 PMC8012333

[9] Hany M, El-Matbouly M, El-Hadidy A, et al. Comparison of the mid-term outcomes of banded and non-banded sleeve gastrectomy: safety, food tolerance, and weight regain. Surg Endosc. 2022;36(12):9146–55. 35764843 10.1007/s00464-022-09395-4 PMC9652222

[10] Reis MG, Santos AL, Silva AM, et al. Weight regain after bariatric surgery: a systematic review and meta-analysis of observational studies. Obes Med. 2024;45:100528.

[11] Shoar S, Khorgami Z, Brethauer SA, et al. Banded versus nonbanded Roux-en-Y gastric bypass: a systematic review and meta-analysis of randomized controlled trials. Surg Obes Relat Dis. 2019;15(5):688–95. 31255232 10.1016/j.soard.2019.02.011

[12] Jense MTF, Palm-Meinders IH, Sigterman-Nelissen R, et al. The benefits of banded over non-banded Roux-en-Y gastric bypass in patients with morbid obesity: a multi-center study. Obes Surg. 2022;32(6):1856–63. 35366739 10.1007/s11695-022-06024-w PMC9072269

[13] van Helmond S, Jense M, Verkoulen G, et al. Pouch Revision in Combination with Placement of a MiniMizer Ring as a Revisional Procedure in Patient with Suboptimal Clinical Response or Recurrent Weight Gain After RYGB. OBES SURG. 2025;35:2990–7. 10.1007/s11695-025-07984-5. 40616624 10.1007/s11695-025-07984-5 PMC12380964

[14] Torensma B, Hany M, Berends F, et al. Clarifying Terminology in Bariatric Metabolic Surgery: The Need for Distinction Between Band and Ring. OBES SURG. 2024;34:1958–9. 10.1007/s11695-024-07168-7. 38499945 10.1007/s11695-024-07168-7

[15] van Dam KAM, de Witte E, Broos PPHL, et al. Short-term safety and effectiveness of conversion from sleeve gastrectomy to ring augmented Roux-en-Y gastric bypass. BMC Surg. 2024;24:266. 10.1186/s12893-024-02552-7. 39300438 10.1186/s12893-024-02552-7 PMC11411827

[16] Bhandari M, Fobi MA, Buchwald JN, et al. Conversion to a banded gastric bypass is a safe and effective option after sleeve gastrectomy: an Indian single-center experience. Obes Surg. 2022;32(1):51.

[17] de Villiers MR, de Villiers PJ, Kent AP. The Delphi technique in health sciences education research. Med Teach. 2005;27(7):639–43. 16332558 10.1080/13611260500069947

[18] Page MJ, McKenzie JE, Bossuyt PM, et al. The PRISMA 2020 statement: an updated guideline for reporting systematic reviews. BMJ. 2021;372:n71. 33782057 10.1136/bmj.n71 PMC8005924

[19] Higgins JP, et al. The Cochrane Collaboration’s tool for assessing risk of bias in randomised trials. BMJ. 2011;343:d5928. 22008217 10.1136/bmj.d5928 PMC3196245

[20] Guyatt GH, et al. GRADE guidelines: 12. Preparing summary of findings tables-binary outcomes. J Clin Epidemiol. 2013;66(2):158–72. 22609141 10.1016/j.jclinepi.2012.01.012

[21] Skidmore A, Holt J, et al. Weight loss and complications of the banded Roux-en-Y gastric bypass: lessons learned from a prospective case control study. Surg Endosc. 2022;36(10):7516–20. 35294635 10.1007/s00464-022-09184-z

[22] Tsai C, Wang W, Li J, et al. Insufficient weight loss after banded vs. non-banded primary gastric bypass surgery: insights from an observational 5 year follow-up study. Surg Endosc. 2022;36(8):5964–69. 34981228 10.1007/s00464-021-08952-7

[23] Ferreira EVB, Santos AL, Silva AM, et al. Long-term comparative evaluation of weight loss and complications of banded and non-banded Roux-en-Y gastric bypass. Obes Surg. 2024;34(8):2923–29. 38884901 10.1007/s11695-024-07354-7

[24] Lemmens L, et al. Banded gastric bypass: better long-term results? A cohort study with minimum 5-year follow-up. Obes Surg. 2017;27:864–72. 27714527 10.1007/s11695-016-2397-4 PMC5339319

[25] Bhandari M, Fobi MA, Buchwald JN, et al. Comparison between banded and nonbanded Roux-en-Y gastric bypass with 2-year follow-up: a preliminary retrospective analysis. Obes Surg. 2016;26:213–18. 26482162 10.1007/s11695-015-1929-7

[26] Awad W, Garay A, Martínez C, et al. Ten years experience of banded gastric bypass: does it make a difference? Obes Surg. 2012;22:271–78. 22161231 10.1007/s11695-011-0555-2

[27] Heneghan HM, Meron-Eldar S, Brethauer SA, et al. Banded Roux-en-Y gastric bypass for the treatment of morbid obesity. Surg Obes Relat Dis. 2014;10(2):210–16. 24462315 10.1016/j.soard.2013.10.016

[28] Jense MTF, Bruinsma FFE, Nienhuijs SW, et al. Ring augmentation of the Roux-en-Y gastric bypass: a propensity score matched analysis of 5-year follow-up results. Obes Surg. 2025;35:884–93. 39883395 10.1007/s11695-025-07706-x PMC11906517

[29] Zarate X, Arceo-Olaiz R, Hernandez JM et al. Long-term results of a randomized trial comparing banded versus standard laparoscopic Roux-en-Y gastric bypass. Surg Obes Relat diseases: official J Am Soc Bariatr Surg 9,3 (2013): 395–7. 10.1016/j.soard.2012.09.009 10.1016/j.soard.2012.09.009 23260801

[30] Pullman JS, Smith BR, Brown JD, et al. Seven-year results of a randomized trial comparing banded Roux-en-Y gastric bypass to sleeve gastrectomy for type 2 diabetes and weight loss. Obes Surg. 2023;33(7):1989–96. 37243915 10.1007/s11695-023-06635-x PMC10224662

[31] Murphy R, Heneghan HM, O’Kane M, et al. Effect of banded Roux-en-Y gastric bypass versus sleeve gastrectomy on diabetes remission at 5 years among patients with obesity and type 2 diabetes: a blinded randomized clinical trial. Diabetes Care. 2022;45(7):1503–11. 35554515 10.2337/dc21-2498 PMC9274222

[32] Murphy R, Heneghan HM, O’Kane M, et al. Laparoscopic sleeve gastrectomy versus banded Roux-en-Y gastric bypass for diabetes and obesity: a prospective randomised double-blind trial. Obes Surg. 2018;28:293–302. 28840525 10.1007/s11695-017-2872-6

[33] Bhandari M, Fobi MA, Buchwald JN, et al. OAGB vs BGBP: a retrospective comparative study of a cohort of patients who had bariatric surgery in 2012 at one centre by a single surgeon. Clin Obes. 2019;9(4):e12308. 30957418 10.1111/cob.12308

[34] Fink JM, Hany M, El-Matbouly M, et al. Banded versus non-banded sleeve gastrectomy: 5-year results of a 3-year randomized controlled trial. Obes Surg. 2024;34(2):310–17. 38109013 10.1007/s11695-023-06982-9 PMC10810940

[35] Hany M, El-Matbouly M, El-Hadidy A, et al. Comparison of sleeve volume between banded and non-banded sleeve gastrectomy: midterm effect on weight and food tolerance—a retrospective study. Obes Surg. 2023;33(2):406–17. 36508154 10.1007/s11695-022-06404-2 PMC9889434

[36] Fink JM, Hany M, El-Matbouly M, et al. Banded versus nonbanded sleeve gastrectomy: a randomized controlled trial with 3 years of follow-up. Ann Surg. 2020;272(5):690–95. 32657920 10.1097/SLA.0000000000004174

[37] Bhandari M, Fobi MA, Buchwald JN, et al. Banded versus nonbanded laparoscopic sleeve gastrectomy: 5-year outcomes. Surg Obes Relat Dis. 2019;15(9):1431–8. 31548001 10.1016/j.soard.2019.04.023

[38] Fink JM, Hany M, El-Matbouly M, et al. Banded versus nonbanded sleeve gastrectomy: 5-year results of a matched-pair analysis. Surg Obes Relat Dis. 2019;15(8):1233–8. 31285129 10.1016/j.soard.2019.05.023

[39] Karcz WK, Karcz-Socha I, Krawczykowski D, et al. To band or not to band—early results of banded sleeve gastrectomy. Obes Surg. 2014;24:660–5. 24464518 10.1007/s11695-014-1189-y

[40] Albalkiny S, El-Matbouly M, El-Hadidy A, et al. The impact of using polytetraflouroethylene (PTFE)-made band in laparoscopic sleeve gastrectomy on the short-and mid-term outcomes of weight loss, a prospective cohort study. Egypt J Surg. 2021;40(4):1095–103.

[41] Salvi P, Fobi MA, Buchwald JN, et al. Banded sleeve gastrectomy and one anastomosis gastric bypass/mini-gastric bypass for treatment of obesity: a retrospective cohort comparative study with 6 years follow-up. Obes Surg. 2020;30:1303–9. 31898044 10.1007/s11695-019-04369-3

[42] Cazzo E, Pareja JC, Chaim EA, et al. Weight loss and vomiting 1 year after banded versus non-banded one anastomosis gastric bypass: a prospective randomized trial. Obes Surg. 2020;30:1719–25. 31942688 10.1007/s11695-020-04393-8

[43] Quint E, El-Matbouly M, El-Hadidy A, et al. Banded One-Anastomosis Gastric Bypass (BOAGB) for patients living with obesity and extreme obesity: a single institution’s experience. Obes Surg. 2024;34(5):1756–63. 38557949 10.1007/s11695-024-07194-5

[44] Elhoofy A, Nagy M, Elghandour AM, et al. Banded One Anastomosis Gastric Bypass versus Non-Banded One Anastomosis Gastric Bypass: A 3-year Prospective Cohort Study. Ain Shams J Surg. 2024;17(4):349–55.

[45] Hany M, El-Matbouly M, El-Hadidy A, et al. Two-year results of the banded versus non-banded re-sleeve gastrectomy as a secondary weight loss procedure after the failure of primary sleeve gastrectomy: a randomized controlled trial. Obes Surg. 2023;33(7):2049–63. 37156932 10.1007/s11695-023-06598-z PMC10166688

[46] Bhandari M, Fobi MA, Buchwald JN, et al. Conversion to a Banded Gastric Bypass is a Safe and Effective Option after Sleeve Gastrectomy: An Indian Single-center Experience. World. 2022;15(1):51.

[47] Boerboom A, Aarts E, Lange V, et al. Banding the Pouch with a Non-adjustable Ring as Revisional Procedure in Patients with Insufficient Results After Roux-en-Y Gastric Bypass: Short-term Outcomes of a Multicenter Cohort Study. Obes Surg. 2020;30:797–803. 31898043 10.1007/s11695-019-04361-x

[48] Pavone G et al. Banded versus non-banded Roux-en-Y gastric bypass: short, mid, and long-term surgical outcomes—a systematic review and meta-analysis. Surg Obes Relat Dis. 2024. 10.1016/j.soard.2024.05.010 10.1016/j.soard.2024.05.010 38960827

[49] Magouliotis DE, Tasiopoulou VS, Svokos KA, et al. Banded vs. non-banded Roux-en-Y gastric bypass for morbid obesity: a systematic review and meta-analysis. Clin Obes. 2018;8(6):424–33. 30144284 10.1111/cob.12274

[50] Chaouch MA, El-Matbouly M, El-Hadidy A, et al. Banded versus non-banded sleeve gastrectomy: A systematic review and meta-analysis. Med (Baltim). 2023;102(15):e32982. 10.1097/MD.0000000000032982 PMC10101294 37058050

[51] Facciorusso A, Del Prete V, Antonino M, et al. Banded versus non-banded sleeve gastrectomy in obese patients: a systematic review and meta-analysis. Am J Surg. 2022;224(4):1156–61. 35623944 10.1016/j.amjsurg.2022.05.015

[52] Al-Juhani A, Sharaf GF, Alyaseen EM, et al. Banded versus non-banded sleeve gastrectomy: a systematic review and meta-analysis. Cureus. 2024;16(1):e52799. 38389592 10.7759/cureus.52799 PMC10883259

[53] Gupta M, Singla V, Kumar A, et al. Banded sleeve gastrectomy vs non-banded sleeve gastrectomy: a systematic review and meta-analysis. Obes Surg. 2022;32(8):2744–52. 35653009 10.1007/s11695-022-06129-2

[54] Corcelles R, Boules M, Froylich D, et al. Total weight loss as the outcome measure of choice after Roux-en-Y gastric bypass. Obes Surg. 2016;26:1794–8. 26803753 10.1007/s11695-015-2022-y

[55] Anderson B, Grantcharov T, Switzer NJ, et al. Weight loss and clinical outcomes following primary versus secondary Roux-en-Y gastric bypass: a multi-institutional experience. Surg Endosc. 2023;37(8):6445–51. 37217683 10.1007/s00464-023-10133-7

[56] Tettero OM, Monpellier VM, Janssen IMC, et al. Early postoperative weight loss predicts weight loss up to 5 years after Roux-en-Y gastric bypass, banded Roux-en-Y gastric bypass, and sleeve gastrectomy. Obes Surg. 2022;32(9):2891–902. 35842505 10.1007/s11695-022-06166-x PMC9392686

