# Correction to: Ring-augmented Metabolic Bariatric Surgery Procedures GRADE-based International Federation for the Surgery of Obesity and Metabolic Disorders (IFSO) Position Statement

**DOI:** 10.1007/s11695-026-08573-w

**Published:** 2026-03-13

**Authors:** Kayleigh Ann Martina van Dam, Maurizio De Luca, Matteo Monami, Evert-Jan Gijsbert Boerma, Giovanni Merola, Antonio Vitiello, Ricardo Cohen, Ashraf Haddad, Khaled Gawdat, Jodok Fink, Mohammad Hayssam El Fawal, Amanda Belluzzi, Jan Willem Greve

**Affiliations:** 1 Department of surgery, Zuyderland Medisch Centrum, Heerlen, Netherlands; 2 Department of surgery, NUTRIM, institute for Nutrition and Translational Research in Metabolism, Maastricht University, Maastricht, Netherlands; 3 Department of Emergency, General, Bariatric and Metabolic Surgery, Ospedale Santa Maria della Misericordia di Rovigo, Rovigo, Italy; 4 Diabetology Unit, Azienda Ospedaliero-Universitaria Careggi, Florence, Italy; 5 San Giovanni di Dio Hospital, Naples, Italy; 6 Department of Advanced Biomedical Sciences, University of Naples Federico II, Naples, Italy; 7 The center for Obesity and Diabetes, Hospital Alemão Oswaldo Cruz, São Paulo, Brazil; 8 Gastrointestinal Metabolic and Bariatric Surgery Center (GBMC), Jordan Hospital, Amman, Jordan; 9 Kafrelsheikh University, Kafr ash Shaykh, Egypt; 10 Centre for Surgery, Department of General and Visceral Surgery, Centre for Obesity and Metabolic Surgery, University of Freiburg, Freiburg, Germany; 11 Makassed General Hospital, Beirut, Lebanon; 12 Department of surgery, NUTRIM, institute for Nutrition and Translational Research in Metabolism, Maastricht University, Maastricht, Netherlands

**Correction to: Obesity Surgery**

10.1007/s11695-026-08556-x

The correct name of the eleventh author is Mohammad Hayssam El Fawal.

